# Human functional genetic studies are biased against the medically most relevant primate-specific genes

**DOI:** 10.1186/1471-2148-10-316

**Published:** 2010-10-20

**Authors:** Lili Hao, Xiaomeng Ge, Haolei Wan, Songnian Hu, Martin J Lercher, Jun Yu, Wei-Hua Chen

**Affiliations:** 1 CAS Key Laboratory of Genome Sciences and Information, Beijing Institute of Genomics, Chinese Academy of Sciences, 100029 Beijing, China; 2 Graduate University of Chinese Academy of Sciences, 100049 Beijing, China; 3 Bioinformatics group, Heinrich-Heine University Düsseldorf, 40225, Germany; 4 European Molecular Biology Laboratory (EMBL), Meyerhofstrasse 1, 69117 Heidelberg, Germany

## Abstract

**Background:**

Many functional, structural and evolutionary features of human genes have been observed to correlate with expression breadth and/or gene age. Here, we systematically explore these correlations.

**Results:**

Gene age and expression breadth are strongly correlated, but contribute independently to the variation of functional, structural and evolutionary features, even when we take account of variation in mRNA expression level. Human genes without orthologs in distant species ('young' genes) tend to be tissue-specific in their expression. As computational inference of gene function often relies on the existence of homologs in other species, and experimental characterization is facilitated by broad and high expression, young, tissue-specific human genes are often the least characterized. At the same time, young genes are most likely to be medically relevant.

**Conclusions:**

Our results indicate that functional characterization of human genes is biased against young, tissue-specific genes that are mostly medically relevant. The biases should not be taken lightly because they may pose serious obstacles to our understanding of the molecular basis of human diseases. Future studies should thus be designed to specifically explore the properties of primate-specific genes.

## Background

Proteins and their encoding genes can be characterized by functional attributes, such as which pathways they act in or what molecular functions they have; structural attributes, such as lengths of their coding regions or UTRs and GC contents; and evolutionary attributes, such as substitution rates between species and estimates of selection pressures. With the increasing availability of functional genomic data and systems biology tools, correlations between some of these attributes have been observed. The two factors with the strongest associations with other data types in humans are expression breadth (the number of tissues one protein is expressed in) and phyletic age (defined by the evolutionarily most distant species where homologs can be found) [1,2]. For example, recent studies have shown that expression breadth correlated with promoter architecture, evolutionary rates (Ka and Ks) and gene length [2,3], while human proteins of different phyletic age are enriched in distinct functional categories [1]. Interestingly, many properties that correlate with expression breadth also correlate with phyletic age and vice versa; indeed, some studies have reported correlations between phyletic age and expression breadth [2,4].

In evolutionary history, proteins involved in basic biological processes such as transcription and translation machineries, metabolism and cell cycle control were probably invented first. Accordingly, the corresponding genes are old, and conserved in multiple organisms, and are expressed in multiple tissues. Conversely, younger genes perform more specialized functions, and are thus typically used and expressed in specific tissues and/or environments. This line of reasoning predicts a significant correlation between phyletic age and expression breadth. Consequently, variables correlate with one of the two factors should also correlate with the other. For example, many old genes are expected to be broadly useful (and hence broadly expressed), to come from basic functional groups (such as transcription/translation or metabolism), and to require distinct promoter architectures and optimized gene structures; they may also evolve mostly under strong purifying selection.

This picture of increasing specialization with decreasing gene age is an oversimplification. For example, gene duplication may often be followed by subfunctionalization [5], where each of the two gene copies takes over the function of the ancestral gene in part of the tissues of the ancestral gene. A recent study suggested that subfunctionalization was indeed partially underlying the correlation between the rate of increase in gene tissue specificity and the rate of increase in the maximum number of cell types [6]. The duplication thus creates two gene copies with the same phyletic age, but very different expression profiles [7,8]; other changes of gene attributes (e.g., sequence changes to optimize tissue-specific function) might follow. Furthermore, genes expressed in different tissues may be under different constraints, resulting in variable evolutionary rates across proteins with similar expression breadth [9]. Another important factor known to influence gene structure and evolution is expression abundance [10-12]. Thus, while we expect a strong overlap between the influence of phyletic age and expression breadth on human protein features, we expect age and breadth to differ in their influence on individual proteins; and we expect that other factors, such as expression abundance, also contribute to systematic variation in protein attributes.

Functional studies of proteins and their encoding genes are biased by gene properties. For example, highly and broadly expressed gene products are easy to detect and are thus studied preferentially. Older genes, which are most likely conserved across multiple species (especially model organisms used for disease models), are studied more intensively due to the availability of animal (or even yeast) models. Proteins with basic molecular functions were studied preferentially because of their importance in biology. Due to these biases, we expect that databases like Gene Ontology [13] and KEGG pathways [14,15] contain more reliable functional annotations for old and broadly expressed genes. Conversely, younger genes, which may be important, e.g., in human-specific gene regulation, are not studied as much.

In this study, we focus on human protein-coding genes to explore systematic correlations of phyletic age and expression profiles with some intrinsic gene features, as well as the interdependence of age and expression breadth. Based on our observations, we discuss systematic biases in functional studies that affect the process of knowledge acquisition for human disease-related genes.

## Results

### If it increases with expression breadth, it probably increases with age

**Figure 1:**
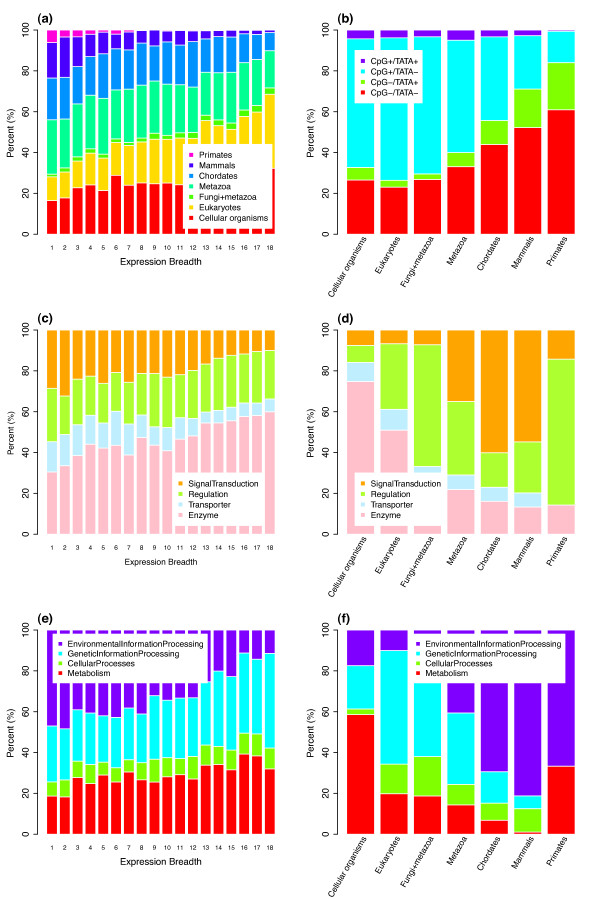
**Correlations of human protein-coding gene properties with expression breadth and phyletic age**. The numbers in the x-axis of panels a, c, e indicate the numbers of tissues in which genes are expressed. Phyletic groups in panels b, d, f are arranged according to their age, with 'cellular organisms' being the oldest and 'primates' the youngest. (a) Broadly expressed human proteins tend to be older, i.e., have homologs in more distantly related species. (b) Genes of different ages have distinct promoter architectures. (c-f) Gene function (according to GO and KEGG annotation) is correlated with both expression breadth (c, e) and phyletic age (d, f).

Using previously published phyletic age classifications [1] and expression breadth data [2,3], we first verified the substantial correlation between evolutionary age and expression breadth in human genes (Pearson's correlation coefficient = 0.27, P < 10-15). Consistent with our hypothesis of a later origin of proteins with more specific functions, we find that older genes are on average more broadly expressed, while young genes tend to be tissue-specific (Figure 1a), tissue-specificity is most pronounced for genes restricted to primates or mammals.

Genes of different expression breadth exhibit different promoter architectures [2,16]. Characterizing promoter architecture through the presence of CpG-islands and TATA-boxes, we confirmed that CpG+/TATA- promoter presence is positively correlated with expression breadth (Pearson's correlation coefficient = 0.394, P < 10-15), while CpG-/TATA+ and CpG-/TATA- promoters show a corresponding negative correlation (Additional file 1, Figure S1a). As expected, we found that phyletic age also correlates significantly with promoter architecture (Pearson's correlation coefficient = 0.217, P < 10-15 for CpG+/TATA-, Table 1). As shown in Figure 1b, younger genes are increasingly in favor of promoters lacking CpG islands; these promoters are significantly enriched especially in primate-specific genes (odds ratio: 8.038, P < 10-15, Fisher's exact test).

**Table 1:** Correlation of human protein-coding gene properties with phyletic age and expression breadth

Category	Property	Phyletic age		Expression breadth	
		
		*R *^a^	*P*	*R *^a^	*P*
	Age	-	-	0.270	< 10^-15^***
Structural					
	Protein length (log)	0.340	< 10^-15^***	0.113	< 10^-15^***
	Exon number	0.290	< 10^-15^***	0.185	< 10^-15^***
	CpG+/TATA- promoter	0.217	< 10^-15^***	0.394	< 10^-15^***
	Length 1^st ^intron (log)	0.103	< 10^-15^***	0.063	6.0×10^-14^***
	Length of 5' UTR (log)	0.029	0.0005***	0.127	< 10^-15^***
	GC content of CDS	-0.110	< 10^-15^***	-0.120	< 10^-15^***
Functional					
	Molecular functions	-0.543	< 10^-15^***	-0.178	< 10^-15^***
	Pathway class	-0.264	< 10^-15^***	-0.025	0.042 *
	Expression level (log)	0.162	< 10^-15^***	0.611	< 10^-15^***
Evolutionary					
	Ka	-0.275	< 10^-15^***	-0.258	< 10^-15^***
					
	Ks	-0.062	1.3 × 10^-11^***	-0.050	8.9 × 10^-09^***
	Ka/Ks	-0.336	< 10^-15^***	-0.274	< 10^-15^***

^a ^Pearson's correlation coefficient. * *P *< 0.05;** *P *< 0.01;*** *P *< 0.001.

Overall, we examined five structural gene properties: length of proteins and 5'-UTRs, number of exons, length of the first exon, and GC content of gene's coding regions. Each structural property correlates significantly with both phyletic age and expression breadth (P ≤ 0.0005 in each case, see Table 1, Additional file 1, Figure S1b-g and Figure S2a-f for details). The strongest correlations are those with protein length, gene length and exon numbers, while 5'-UTR length and GC content show weaker correlations. Of particular interest are the weak but statistically highly significant correlations of the length of first introns with phyletic age and expression breadth (R = 0.10 and 0.06, respectively). First introns are known to often harbor regulatory elements [17]. Thus, the results in Table 1 suggest that tissue-specific gene expression is at least in part achieved through additional regulation in 5'-UTRs and first introns. This notion is consistent with the correlation between 5'-UTR length and first intron length (R = 0.095, P < 10-15).

We also examined gene-specific evolutionary rates, estimated from the fraction of synonymous (Ks) and non-synonymous (Ka) sites with nucleotide substitutions between human and mouse orthologs. Ka, Ks, and their ratio Ka/Ks all correlated negatively with both phyletic age and expression breadth, which is consistent with previous findings [1,16] (see Table 1 and Additional file 1, Figure S1h and S2g for details).

Are more ancient human genes really involved in more basal cellular functions? While the relationship between functional categories and phyletic age has been investigated previously [1,4], the trend among human proteins was relatively weak [1]. Here, we used annotation data from two sources, GeneOntology (GO) [13] and pathway annotations from KEGG [14,15]. Of the 22,165 human non-redundant protein-coding genes in this study, 7,452 had GO annotations supported by at least one of the experimental evidence codes (IDA, IPI, IMP, IGI, and IEP). Using a modified method from Freilich et al. [4], we grouped genes into four main categories according to their GO annotation: enzymatic activity, transporter, regulation (including transcription regulator activity, translation regulator activity, and enzyme regulator activity), and signal transduction. These functional annotations indeed correlated with expression breadth (Figure 1c): the fraction of enzymes increases with increasing expression breadth, while the fraction of genes involved in signal transduction decreases. In contrast, the fractions of transporters and genes involved in regulation remained roughly constant across different expression breadths.

While we also found global patterns in the distribution of the functional groups across different age groups, these were not a simple mirror image of the results for expression breadth. Similar to the trend with increasing expression breadth, we found that the combined fraction of enzymes and transporters decreases with phyletic age, while the combined relative number of genes involved in regulation and signal transduction increases (Figure 1d). For expression breadth these trends are almost entirely due to variation in enzyme and regulator fractions. For phyletic age, there is also a decrease in the fraction of transporters in primate-specific genes. Even more strikingly, regulatory genes show a U-shaped distribution, compensated by massively increased fractions of signaling genes in metazoa-, chordata-, and mammalian-specific genes (Figure 1d).

We found a comparable number of our genes (8,569) to be annotated in at least one of four pathway categories in KEGG: metabolism, genetic information processing, environmental information processing, and cellular processes. As shown in Figure 1e and 1f, global trends are very similar to the GO results. However, metabolism appears almost absent among mammalian-specific genes, while environmental information processing is the dominant category also in primate-specific genes. After examining a wide range of structural, evolutionary, and functional properties of human genes, we thus conclude that any trends with increasing expression breadth seem to be always mirrored by according trends with increasing phyletic age, and vice versa.

### Age and expression breadth affect gene properties independently

Are the effects of age and expression pattern independent? Or is one of the two factors responsible for most of the correlations, and the effect of the other factor just due to the mutual relationship between age and expression? And do other factors contribute significantly to the variation of human protein properties? Using a generalized linear model, we find that both age and expression breadth contribute independently to all characteristics of human proteins tested here (Table 2). We also find that mRNA expression abundance contributes significantly to some but not all properties. Interestingly, expression level is correlated with expression breadth [18], but not with phyletic age; under our hypothesis, the observed result suggests that ancient cellular functions do not generally require large protein numbers, but that expression levels are rather driven by tissue-specific effects [9].

**Table 2:** Influence of expression breadth, phyletic age and expression abundance on protein properties using generalized linear model^a^

Category	Property	*P*(phyletic age)	*P*(expression breadth)	*P*(expression abundance)
Structural				
	Protein length (log)	< 10^-15^***	4.7 × 10^-13^***	< 10^-15^***
				
	Exon number	< 10^-15^***	< 10^-15^***	5.50 × 10^-13^***
	CpG+/TATA- promoter	2.30 × 10^-14^***	< 10^-15^***	0.0405*
	1^st ^intron length (log)	0.0061**	0.0240 *	4.43 × 10^-05^***
	Length 5' UTR (log)	0.00747**	< 10^-15^***	4.31 × 10^-05^***
	GC of CDS	< 10^-15^***	< 10^-15^***	8.34 × 10^-11^***
Functional				
	Molecular function (GO)	< 10^-15^***	< 10^-15^***	0.0739
	Pathway class (KEGG)	< 10^-15^***	7.17 × 10^-6^***	0.0121*
Evolutionary				
	Ka	< 10^-15^***	< 10^-15^***	0.180
				
	Ks	7.84 × 10^-5^***	2.41 × 10^-7^***	0.428
	Ka/Ks	< 10^-15^***	< 10^-15^***	0.000354***
MainFactors				
	Age	-	< 10^-15^***	0.348
	EST breadth	< 10^-15^***	-	< 10^-15^***
	Expression abundance	0.348	< 10^-15^***	-

Numbers are the corresponding P-values, * *P *< 0.05;** *P *< 0.01;*** *P *< 0.001.

^a ^We used a form like 'property ~ phyletic age + expression breadth + expression abundance' in the generalized linear model analysis. This form will produce three p-values showing the influence of phyletic age, expression breadth and expression abundance on 'property' respectively; p-values less than certain threshold (0.05 for example) suggest significant contribution of some factors to the 'property'; multiple significant p-values suggest the corresponding factors contribute independently to the 'property'.

### Human functional studies are biased against disease-related genes

**Figure 2:**
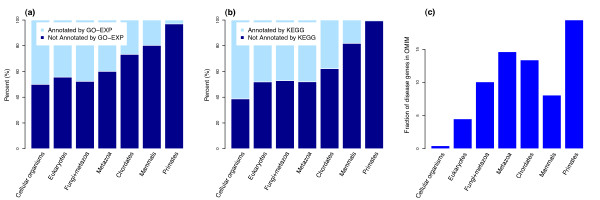
**Young genes are rarely functionally characterized; but the youngest group is the most disease-related**. (a) genes annotated by gene ontology with experimental evidence codes (GO-EXP); (b) genes annotated by KEGG; (c) disease-causing genes annotated by OMIM (Online Mendelian Inheritance in Man).

The most striking trend with decreasing age is that more and more human genes are not annotated in GO or KEGG (Figure 2a and 2b). Most strikingly, the majority of genes in the primate group are functionally uncharacterized. There is a corresponding trend for genes with lower expression breadth to be less annotated in GO (Additional file 1, Figure S1i). Thus, broadly expressed and, in particular, older genes are relatively well studied, while many young, tissue-specific genes are poorly characterized. Interestingly, there is no corresponding increase in the fraction of genes annotated in KEGG with increasing expression breadth (P = 0.28). Thus, annotations in KEGG, which are mostly based on biochemical experiments, appear less biased towards broadly expressed proteins compared with the more heterogeneous GO database.

These biases are not surprising: regularly, the first step in functional annotation is comparison to characterized genes with similar amino acid sequence. The probability of a good match obviously increases with the phylogenetic distribution of homologs; in particular, this approach will seldom be successful for genes that are restricted to primates. Experimental characterization is usually done in model organisms, with a substantial part of our knowledge derived from species as distantly related to humans as insects, nematode worms, or even yeast. Finally, tissue-specific (and often lowly expressed) genes are harder to observe experimentally, adding a bias towards broadly expressed proteins to the phylogenetic bias.

Should we care about this bias? An analysis of disease-related genes in the Online Mendelian Inheritance in Man database (OMIM) [19] shows that such medically relevant genes are strongly enriched among primate-specific genes: about 20% of all genes restricted to humans and other primates are currently associated with diseases (Figure 2c), more than in any other age class; this percentage is very likely an underestimate. That this medically most relevant group of genes is the one least characterized appears problematic.

## Discussion

Grouping human protein-coding genes by both phyletic age and by expression profiles, we observed significant correlations of these two factors with a number of gene properties, including gene structure, GC content, promoter architecture, evolutionary rate, and functional classification. While several of these correlations had been observed previously [1,2,4], we show that in each case both factors, age and expression pattern, independently contribute to the observed variation. The trends are gradual, with substantial overlap between neighboring groups.

Another important gene property correlated with phyletic age as well as expression breadth is the probability of being annotated in public databases (Figure 2a, b and Additional file 1, Figure S1i): the youngest, primate-specific genes are the least characterized functionally, partly because of a lack of orthologs in model species, partly because of the typical gene properties analyzed above. Thus, there is a substantial bias against the functional characterization of certain genes: functional studies preferentially target genes that are conserved in other species, with broad expression breadth and basic functions, while younger genes with temporally and spatially restricted expression are less understood. These biases are not unexpected, as lowly expressed genes are difficult to capture even using high-throughput technologies, and as functional studies are facilitated by the availability of orthologs in model species.

Unfortunately, it is the primate-specific genes that contain the highest fraction of disease-related genes (19.4%, Figure 2c). The systematic biases against their study should thus not be taken lightly: the incomplete functional characterization of the youngest genes may pose serious obstacles to our understanding of the molecular basis of human diseases. Cell line culturing, tissue engineering and the emerging induced pluripotent stem cells technique [20] may help to provide solutions to this bias.

Our results differ markedly from those of Domazet-Loso and Tautz [21,22], which found that the percentage of human disease-related sequences remained approximately constant up to the divergence of the mammalian lineages, and then decreased steeply with decreasing age. One major methodological difference between our study and [21,22] is that these authors identified phylogenetic sequence age based on a very lenient Blast E-value cutoff (0.001). This approach places all gene families that share a particular protein domain into the age class where this domain emerged first, even though a particular gene may have evolved later, e.g., via gene duplication [21,22]. The youngest sequences in [21,22] are thus genes without any recognizable homology to genes in other phyla; it is conceivable that some of these may in fact be mis-annotations. In contrast, the method employed here [1] is designed to confidently assign phylogenetic ages to genes rather than domains. The youngest genes in our study often share protein domains with older sequences, but arose recently by gene duplication or exon shuffling. Thus, while the results of [21,22] apply to the age distribution of disease-associated protein domains, our results give the age distribution of complete disease-associated genes.

## Methods

### Human protein coding genes and gene properties

22,165 non-redundant human protein-coding genes were downloaded from the HomoloGene database release 64 http://www.ncbi.nlm.nih.gov/homologene. Properties of the human genes, including number of exons, intron length, and 3' and 5' UTR length, were calculated based on BLAT mapping results of nucleotide gene sequences to the human genome available at the UCSC Genome Browser [23]. Evolutionary rate of human and mouse orthologs, promoter type, as well as expression breadth of human genes were obtained from [2,3]. Four types of promoter architectures were identified according to the presence and absence of CpG-island and TATA-box in the core promoter [2]. mRNA expression levels across 12 diverse human tissues based on deep sequencing of cDNA fragments were downloaded from the NCBI GEO database [24]. Gene expression (mRNA abundance) was pre-calculated as reads per kilobase of exon model per million uniquely mapped reads (RPKM), and deposited under GEO accession GSE12946 [25].

### Phyletic age

Phyletic ages of all non-redundant protein-coding genes were obtained from [1].

### GO and KEGG pathway annotations and functional classification

Human GO annotations were obtained from the Gene Ontology [13] website http://www.geneontology.org; Only GO annotations with any of the experimental evidence codes IDA, IPI, IMP, IGI or IEP (GO-EXP) were used in this study. Based on GO, human protein-coding genes were divided into four groups: enzyme, transporter, regulation and signal transduction, using a method similar to [4].

Pathway annotations of human genes were obtained from KEGG [14,15]. According to these annotations, human genes were classified into one of five categories: metabolism, genetic information processing, environmental information processing, cellular processes, and human disease. We did not consider the category "human disease", as only a few genes were classified into this category by KEGG.

### Diseases related genes

The 'genemap' file containing a list of the best-curated disease genes was downloaded from the Online Mendelian Inheritance in Man (OMIM) database [19] on April 27, 2010. Out of 12,489 entries, we selected 2,428 entries with the "(3)" tag, for which there is strong evidence that at least one mutation in the particular gene is causative for the disease. In total, 2,349 genes in the gene set analysed in this study were marked as disease-related genes.

## Competing interests

The authors declare that they have no competing interests.

## Authors' contributions

WHC, MJL, JY and SH conceived of this study. LH, WHC, XG, HW controlled and analyzed the data. WHC and MJL wrote the manuscript. All authors read and approved the manuscript.

## Supplementary Material

Additional file 1 **This additional file contains two supplementary figures with corresponding figure legends**. Click here for file

## References

[B1] Wolf YI Novichkov PS Karev GP Koonin EV Lipman DJ Inaugural Article: The universal distribution of evolutionary rates of genes and distinct characteristics of eukaryotic genes of different apparent ages Proc Natl Acad Sci USA 2009 106 18 7273 7280 10.1073/pnas.0901808106 19351897 PMC2666616

[B2] Zhu J He F Hu S Yu J On the nature of human housekeeping genes Trends Genet 2008 24 10 481 484 10.1016/j.tig.2008.08.004 18786740

[B3] Zhu J He F Song S Wang J Yu J How many human genes can be defined as housekeeping with current expression data? BMC Genomics 2008 9 172 10.1186/1471-2164-9-172 18416810 PMC2396180

[B4] Freilich S Massingham T Bhattacharyya S Ponsting H Lyons PA Freeman TC Thornton JM Relationship between the tissue-specificity of mouse gene expression and the evolutionary origin and function of the proteins Genome biology 2005 6 7 R56 10.1186/gb-2005-6-7-r56 15998445 PMC1175987

[B5] Lynch M Force A The Probability of Duplicate Gene Preservation by Subfunctionalization Genetics 2000 154 1 459 473 10629003 10.1093/genetics/154.1.459 PMC1460895

[B6] Milinkovitch MC Helaers R Tzika AC Historical constraints on vertebrate genome evolution Genome Biol Evol 2010 2 13 18 10.1093/gbe/evp052 PMC2839353 20333219

[B7] Gu Z Nicolae D Lu HH Li WH Rapid divergence in expression between duplicate genes inferred from microarray data Trends Genet 2002 18 12 609 613 10.1016/S0168-9525(02)02837-8 12446139

[B8] Adams KL Cronn R Percifield R Wendel JF Genes duplicated by polyploidy show unequal contributions to the transcriptome and organ-specific reciprocal silencing Proceedings of the National Academy of Sciences of the United States of America 2003 100 8 4649 4654 10.1073/pnas.0630618100 12665616 PMC153610

[B9] Su Z Huang Y Gu X Tissue-driven hypothesis with Gene Ontology (GO) analysis Annals of biomedical engineering 2007 35 6 1088 1094 10.1007/s10439-007-9269-y 17372837

[B10] Carmel L Koonin EV A Universal Nonmonotonic Relationship between Gene Compactness and Expression Levels in Multicellular Eukaryotes Genome Biol Evol 2009 2009 0 382 390 10.1093/gbe/evp038 PMC2817431 20333206

[B11] Pal C Papp B Hurst LD Highly expressed genes in yeast evolve slowly Genetics 2001 158 2 927 931 11430355 10.1093/genetics/158.2.927 PMC1461684

[B12] Pal C Papp B Lercher MJ An integrated view of protein evolution Nat Rev Genet 2006 7 5 337 348 10.1038/nrg1838 16619049

[B13] Ashburner M Ball CA Blake JA Botstein D Butler H Cherry JM Davis AP Dolinski K Dwight SS Eppig JT Gene ontology: tool for the unification of biology. The Gene Ontology Consortium Nat Genet 2000 25 1 25 29 10.1038/75556 10802651 PMC3037419

[B14] Kanehisa M Goto S Furumichi M Tanabe M Hirakawa M KEGG for representation and analysis of molecular networks involving diseases and drugs Nucleic Acids Res 2009 38 Database D355 360 19880382 10.1093/nar/gkp896 PMC2808910

[B15] Kanehisa M Goto S KEGG: kyoto encyclopedia of genes and genomes Nucleic Acids Res 2000 28 1 27 30 10.1093/nar/28.1.27 10592173 PMC102409

[B16] Schug J Schuller WP Kappen C Salbaum JM Bucan M Stoeckert CJ Jr Promoter features related to tissue specificity as measured by Shannon entropy Genome biology 2005 6 4 R33 10.1186/gb-2005-6-4-r33 15833120 PMC1088961

[B17] Yoshiro N Tsukasa N Satoshi S Takeshi M Shinji K Takeo K Transcriptional regulatory elements in the 5' upstream and first intron regions of the human smooth muscle (aortic type) [alpha]-actin-encoding gene Gene 1991 99 2 285 289 10.1016/0378-1119(91)90140-7 2022339

[B18] Pal C Papp B Lercher MJ Adaptive evolution of bacterial metabolic networks by horizontal gene transfer Nature genetics 2005 37 12 1372 1375 10.1038/ng1686 16311593

[B19] McKusick VA Mendelian Inheritance in Man and its online version, OMIM Am J Hum Genet 2007 80 4 588 604 10.1086/514346 17357067 PMC1852721

[B20] Takahashi K Tanabe K Ohnuki M Narita M Ichisaka T Tomoda K Yamanaka S Induction of pluripotent stem cells from adult human fibroblasts by defined factors Cell 2007 131 5 861 872 10.1016/j.cell.2007.11.019 18035408

[B21] Domazet-Loso T Tautz D An ancient evolutionary origin of genes associated with human genetic diseases Molecular biology and evolution 2008 25 12 2699 2707 10.1093/molbev/msn214 18820252 PMC2582983

[B22] Domazet-Loso T Tautz D Phylostratigraphic tracking of cancer genes suggests a link to the emergence of multicellularity in metazoa BMC Biol 2010 8 66 10.1186/1741-7007-8-66 20492640 PMC2880965

[B23] Rhead B Karolchik D Kuhn RM Hinrichs AS Zweig AS Fujita PA Diekhans M Smith KE Rosenbloom KR Raney BJ The UCSC Genome Browser database: update 2010 Nucleic Acids Res 2010 38 Database D613 619 10.1093/nar/gkp939 19906737 PMC2808870

[B24] Barrett T Troup DB Wilhite SE Ledoux P Rudnev D Evangelista C Kim IF Soboleva A Tomashevsky M Marshall KA NCBI GEO: archive for high-throughput functional genomic data Nucleic Acids Res 2009 37 Database D885 890 10.1093/nar/gkn764 18940857 PMC2686538

[B25] Wang ET Sandberg R Luo S Khrebtukova I Zhang L Mayr C Kingsmore SF Schroth GP Burge CB Alternative isoform regulation in human tissue transcriptomes Nature 2008 456 7221 470 476 10.1038/nature07509 18978772 PMC2593745

