# Supplementary material for: Human functional genetic studies are biased against the medically most relevant primate-specific genes
Source: BMC Evol Biol. 2010 Oct 20;10:316. doi: 10.1186/1471-2148-10-316 (PMC2970608; doi:10.1186/1471-2148-10-316)
Supplement: Additional file 1 — This additional file contains two supplementary figures with corresponding figure legends. [file 1471-2148-10-316-S1.PDF]

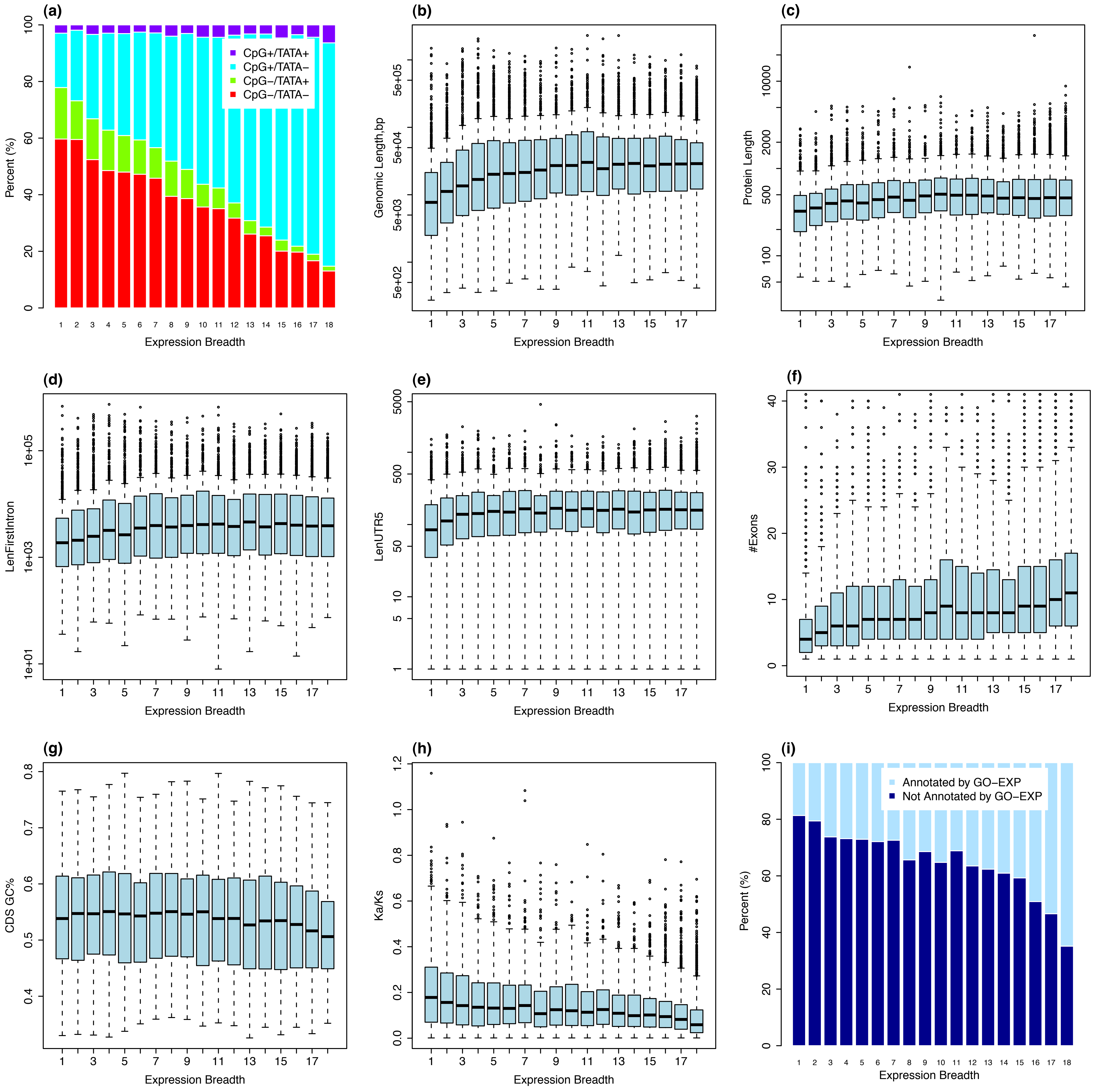

Figure S1. Relationship between expression breadth and other properties of human protein coding genes. (a) promoter architectures, (b) genomic length, (c) protein length, (d) length of first intron, (e) length of 5′ UTR, (f) number of exon, (g) GC content of CDS region, (h) Ka/Ks ratio, (i) fractions of genes annotated by GO.

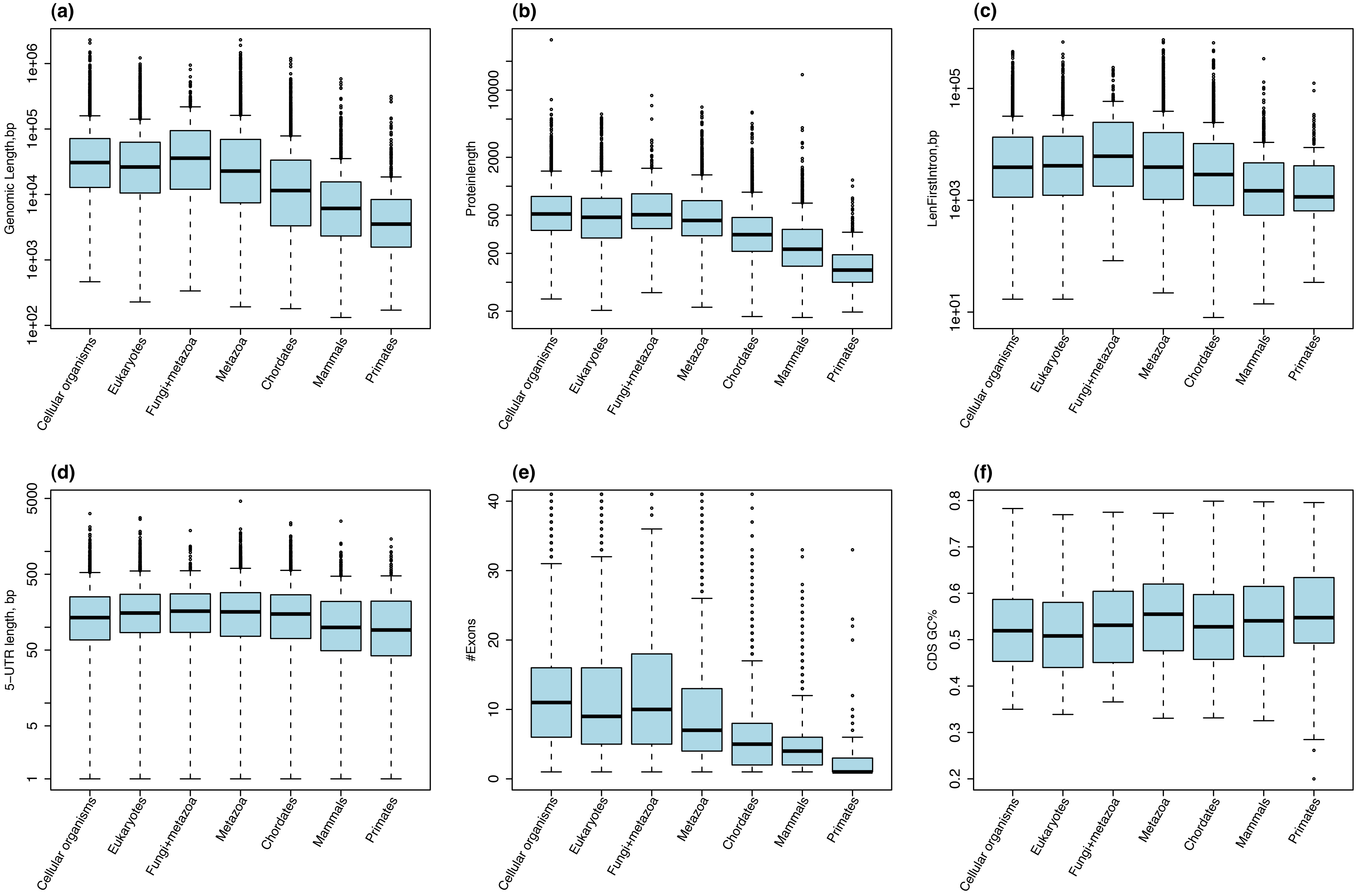

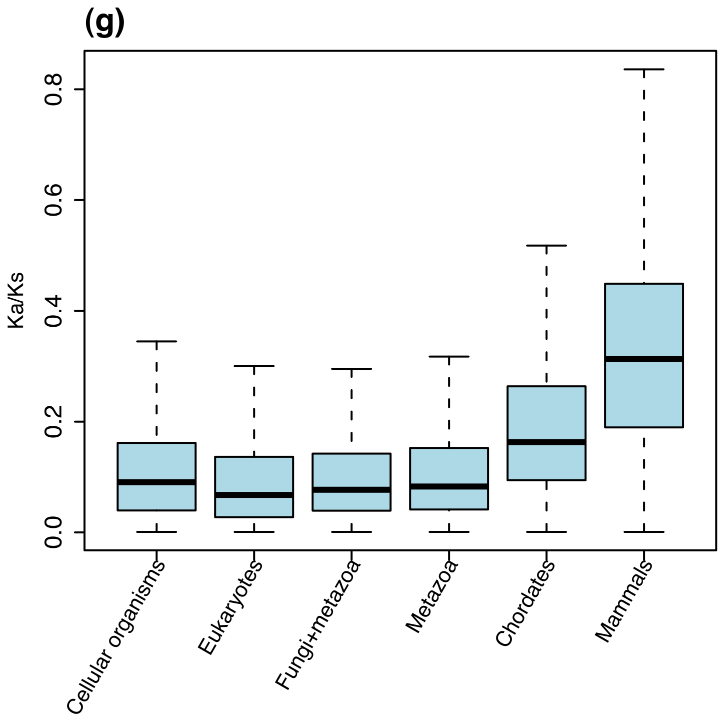

Figure S2. Relationship between phyletic age and other properties of human protein coding genes. (a) genomic length, (b) protein length, (c) length of first intron, (d) length of 5′ UTR, (e) number of exon, (f) GC content of CDS region and (g) Ka/Ks ratio.
